# Acute Effects of Dietary Protein Consumption on the Postprandial Metabolic Response, Amino Acid Levels and Circulating MicroRNAs in Patients with Obesity and Insulin Resistance

**DOI:** 10.3390/ijms25147716

**Published:** 2024-07-14

**Authors:** Karla G. Hernández-Gómez, Laura A. Velázquez-Villegas, Omar Granados-Portillo, Azalia Avila-Nava, Luis E. González-Salazar, Aurora E. Serralde-Zúñiga, Berenice Palacios-González, Edgar Pichardo-Ontiveros, Rocio Guizar-Heredia, Adriana M. López-Barradas, Mónica Sánchez-Tapia, Violeta Larios-Serrato, Viridiana Olin-Sandoval, Andrea Díaz-Villaseñor, Isabel Medina-Vera, Lilia G. Noriega, Gabriela Alemán-Escondrillas, Victor M. Ortiz-Ortega, Nimbe Torres, Armando R. Tovar, Martha Guevara-Cruz

**Affiliations:** 1 Departamento de Fisiología de la Nutrición, Instituto Nacional de Ciencias Médicas y Nutrición Salvador Zubirán, Mexico City 14080, Mexico; 2 Hospital Regional de Alta Especialidad de la Península de Yucatán, IMSS-Bienestar, Mérida 97130, Yucatán, Mexico; 3 Servicio de Nutriología Clínica, Instituto Nacional de Ciencias Médicas y Nutrición Salvador Zubirán, Mexico City 14080, Mexico; 4 Laboratorio de Envejecimiento Saludable del INMEGEN en el Centro de Investigación Sobre el Envejecimiento, Mexico City 14330, Mexico; 5 Instituto Politécnico Nacional, Escuela Nacional de Ciencias Biológicas, Mexico City 11340, Mexico; 6 Departamento de Biotecnología y Bioingeniería, Centro de Investigación y de Estudios Avanzados, Instituto Politécnico Nacional, Mexico City 07360, Mexico; 7 Departamento de Medicina Genómica y Toxicología Ambiental, Instituto de Investigaciones Biomédicas, UNAM, Mexico City 04510, Mexico; 8 Departamento de Metodología de la Investigación, Instituto Nacional de Pediatría, Mexico City 04530, Mexico

**Keywords:** dietary protein, insulin resistance, obesity, amino acids, miRNAs

## Abstract

The post-nutritional intervention modulation of miRNA expression has been previously investigated; however, post-acute dietary-ingestion-related miRNA expression dynamics in individuals with obesity and insulin resistance (IR) are unknown. We aimed to determine the acute effects of protein ingestion from different dietary sources on the postprandial metabolic response, amino acid levels, and circulating miRNA expression in adults with obesity and IR. This clinical trial included adults with obesity and IR who consumed (1) animal-source protein (AP; calcium caseinate) or (2) vegetable-source protein (VP; soy protein isolate). Glycaemic, insulinaemic, and glucagon responses, amino acid levels, and exosomal microRNAs isolated from plasma were analysed. Post-AP ingestion, the area under the curve (AUC) of insulin (*p* = 0.04) and the plasma concentrations of branched-chain (*p* = 0.007) and gluconeogenic (*p* = 0.01) amino acids increased. The effects of different types of proteins on the concentration of miRNAs were evaluated by measuring their plasma circulating levels. Compared with the baseline, the AP group presented increased circulating levels of miR-27a-3p, miR-29b-3p, and miR-122-5p (*p* < 0.05). Subsequent analysis over time at 0, 30, and 60 min revealed the same pattern and differences between treatments. We demonstrated that a single dose of dietary protein has acute effects on hormonal and metabolic regulation and increases exosomal miRNA expression in individuals with obesity and IR.

## 1. Introduction

Obesity is a risk factor for the development of cardiovascular and metabolic diseases, which are among the leading causes of death worldwide [1]. One of these obesity-related conditions is insulin resistance (IR), which is a key metabolic condition in the pathophysiology of type 2 diabetes mellitus (T2DM) [2]. IR is reversible with adequate and timely treatment, and nutritional treatment plays a fundamental role [3]. Different strategies focused on reducing both obesity and IR have been developed; dietary strategies are proposed first, with the objective of changing habits and lifestyles [4]. One of the most common strategies is energy restriction; however, all three macronutrients are usually restricted. While reductions in lipid and carbohydrate intake are associated with decreased metabolic disturbances, this association has not been well established with protein intake [5]. Proteins are known to play a key role in the regulation of metabolic factors, such as insulin signalling [6]. This regulation is due to the type of amino acid that comprises the protein, particularly the branched-chain amino acids (BCAAs) valine, isoleucine and leucine. Studies have shown that these amino acids are associated with IR and, thus, an increased risk of T2DM [7]. Therefore, considering the type of protein ingested is important since, compared with animal proteins, vegetable protein contains lower levels of BCAAs [8]. Therefore, regulating the type of dietary protein may represent an important nutritional strategy for modulating plasma levels of circulating amino acids, particularly BCAAs [9].

Metabolic modulation through dietary interventions can also occur through mechanisms regulated at different time points that involve various molecules, such as microRNAs (miRNAs). miRNA expression can be affected by multiple factors, such as nutritional or pathological status, including obesity. Variations in circulating miRNAs have been detected under conditions of obesity, IR and subsequent dietary intervention, and twelve miRNAs, including miR-Let-7, miR-20, miR-21, miR-27, miR-29, miR-34, miR-99, miR-122, miR-126, miR-143, miR-222, and miR-223, converge under these three conditions [10]. This result highlights the importance of miRNAs as potential predictive biomarkers of the dietary response, the importance of understanding the mechanisms underlying acute physiological responses, and how miRNA-induced transcriptional repression of target mRNAs is implicated in the pathophysiology of obesity and IR [11]. This evidence shows that molecular regulation under pathological and nutritional conditions can be modulated in response to different dietary treatments through the expression of miRNAs. Unfortunately, few clinical studies have investigated this interaction, but our review highlights the work of Mantilla-Escalante et al., which includes overweight and obese subjects subjected to dietary interventions. In this study, they identified exosomal miRNAs associated with insulin resistance and modified their expression after one year of dietary intervention [12]. They noted that differentially expressed miRNAs, in turn, regulate various genes involved in metabolic pathways, such as insulin signalling, participating in the development of insulin resistance [12].

In this study, seven circulating miRNAs were identified that coincided with those identified in our review; a second analysis of those miRNAs that had the greatest support in the literature for effects on their circulating levels after a dietary intervention revealed that four miRNAs (miR-27a-3p, miR-29b-3p, miR-122-5p, and miR-222-3p) among the seven candidates could help us develop an initial approach to assess the dynamics of their acute regulation and contribute to fulfilling the objectives of our study.

Notably, miR-27a-3p, miR-29b-3p, miR-122-5p, and miR-222-3p have received considerable attention because of their roles in regulating genes involved in metabolic pathways, such as insulin, lipid metabolism, glucose metabolism or inflammatory processes. The support in the literature is extensive. An animal study revealed increased circulating levels of miRNAs, such as miR-122, miR-192, miR-27a-3p, and miR-27b-3p, in a model of obesity. They isolated exosomes from obese model mice and used them to treat mice without obesity, observing that glucose intolerance and insulin resistance were induced in mice without obesity [13].

Among the few studies on the levels of miRNAs and their associations with metabolic diseases, we highlight the work performed in Mayan school children with and without obesity, in which it was found that circulating levels of miR-122 and miR-222 were 1.47 and 1.78 times higher, respectively, in obese children. In addition, elevated levels of miR-122 and miR-222 were associated with BMI, the waist–to–height ratio, fat percentage, HDL cholesterol levels, TG levels and the metabolic index (MI), and the authors reported that high circulating levels of miR-122 and miR-222 conferred an increased risk of developing obesity [14]. Another study of subjects classified as overweight or obese, metabolically healthy or metabolically unhealthy, revealed that elevated circulating levels of miR-221, miR-21, and miR-29 were present in the metabolically unhealthy overweight and obese groups, which correlated with the levels of leptin and proinflammatory interleukins, and the authors also reported an increased expression of SIRT1 mRNA and decreased expression of PGC1α mRNA in these patients, suggesting decreased fatty acid oxidation metabolism [15]. For these reasons, the aim of this study was to evaluate the responses of insulin, glucagon and amino acid profiles in plasma and the relative expression of circulating miRNAs miR-27a-3p, miR-29b-3p, miR-122-5p, and miR-222-3p in adults with obesity and IR after acute experimental stimulation with animal-source protein (calcium caseinate) or plant-source protein (soy protein isolate) consumption.

## 2. Results

### 2.1. Anthropometric, Clinical, and Biochemical Parameters

Ten subjects were included, of whom 60% were female (*n* = 6), with a median age of 33.0 years (interquartile range (IQR 27.5, 43.2) and a BMI of 36.8 years (IQR 31.8, 41.3). The general characteristics and biochemical parameters are shown in Table S1.

### 2.2. Areas under the Curves of Insulin, Glucose, and Glucagon in Response to the Intervention

**Figure 1:**
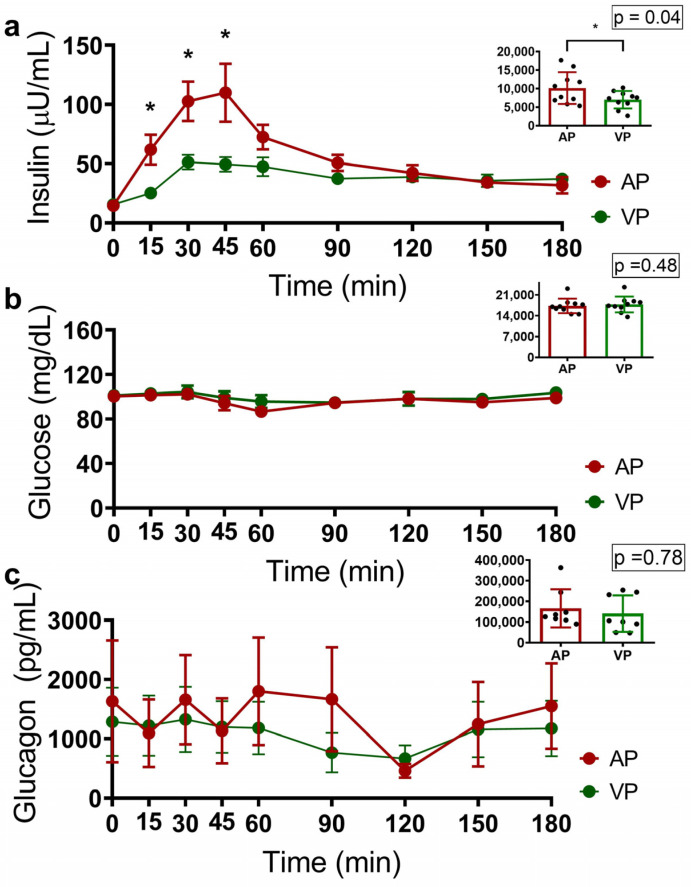
Acute insulin (**a**), glucose (**b**), and glucagon (**c**) responses 3 h after consuming a protein shake containing calcium caseinate (AP—red line) or soy protein isolate (VP—green line), and the areas under the curves (AUCs); the data are presented as the means ± SEs, and the results of the statistical analysis were evaluated using the Mann–Whitney U test. where * *p* < 0.05.

Glucose, insulin, and glucagon curves were generated to evaluate the metabolic effects of the different types of protein. The group that consumed AP had higher insulin concentrations at 15, 30, and 45 min (*p* = 0.013, *p* = 0.010 and *p* = 0.028, respectively), as did the group that consumed VP (*p* = 0.04), as well as a greater area under the curve (AUC) (Figure 1a). No differences were observed in glucose or glucagon concentrations or in the AUCs (Figure 1b,c).

### 2.3. Area under the Amino Acid Curve in Response to the Intervention

**Figure 2:**
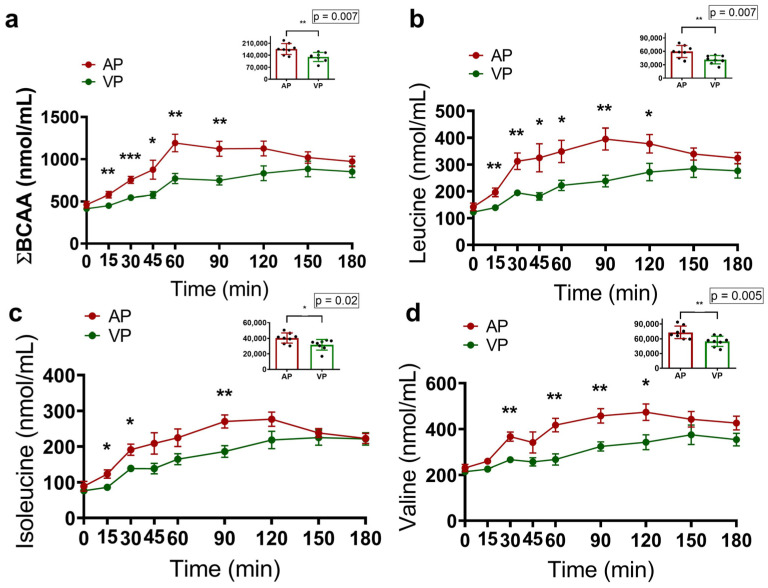
Acute response of amino acids grouped into branched-chain amino acids (BCAAs) (**a**) and their constituents, leucine (**b**), isoleucine (**c**), and valine (**d**), 3 h after consuming a protein shake made with calcium caseinate (AP) or soy protein isolate (VP), and the areas under the curves (AUCs); data are presented as the means ± SEs, and the statistical analysis was evaluated using the Mann–Whitney U test, where * *p* < 0.05, ** *p* < 0.01, and *** *p* < 0.001.

The effect on protein metabolism was evaluated by measuring the concentrations of circulating amino acids. The AP group had higher AUCs for valine (*p* = 0.005), isoleucine (*p* = 0.02) and leucine (*p* = 0.007) (Figure 2b–d). Additionally, lysine (*p* = 0.05), proline (*p* = 0.005) and tyrosine (*p* = 0.007) levels were higher than those in the VP group (Figure S1). Although no differences were detected in the AUCs of glycine, phenylalanine, alanine, arginine, asparagine, aspartate, glutamate, glutamine, serine, histidine, methionine, threonine, or tryptophan, differences in the specific times of the curves were detected for these amino acids (Figures S1 and S2).

**Figure 3:**
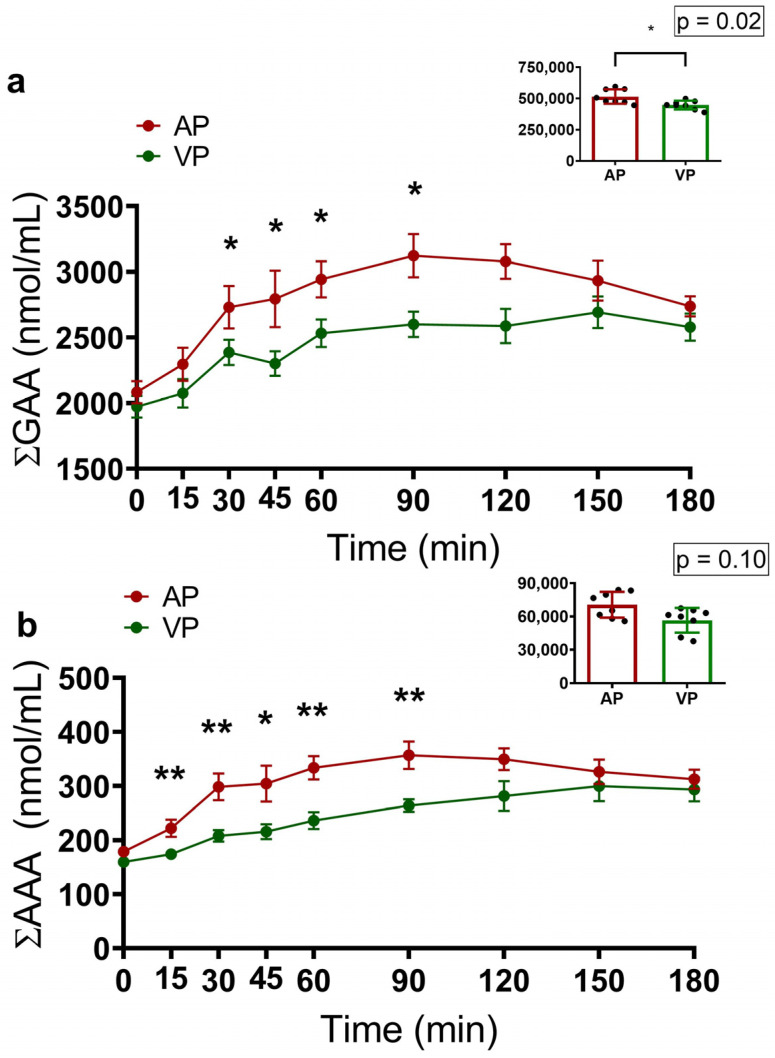
Acute responses of amino acids grouped into gluconeogenic amino acids (∑GAA, (**a**)) and aromatic amino acids (∑AAA, (**b**)) 3 h after consuming a protein shake made with calcium caseinate (PA) or soy protein isolate (PV), and the areas under the curves (AUCs); data are presented as the means ± SEs, and the statistical analysis was evaluated via the Mann–Whitney U test. where * *p* < 0.05 and ** *p* < 0.01.

The analysis of ∑BCAAs revealed that the AP group had higher AUC values (*p* = 0.007) and concentrations of these amino acids at 15 (*p* = 0.005), 30 (*p* = 0.001), 45 (*p* = 0.05), 60 (*p* = 0.01), and 90 (*p* = 0.001) minutes (Figure 2). According to the analysis of gluconeogenic amino acids (∑GAA), the AP group presented increases in AUCs (*p* = 0.02) and the circulating concentrations of these amino acids at 15 (*p* = 0.03), 30 (*p* = 0.05), 45 (*p* = 0.02), and 90 (*p* = 0.01) minutes (Figure 3a).

The concentrations of aromatic amino acids (∑AAAs) in the AP group increased at 15 (*p* = 0.003), 30 (*p* = 0.005), 45 (*p* = 0.05), 60 (*p* = 0.005), and 90 (*p* = 0.007) minutes. However, no significant difference in the AUC was observed (*p* = 0.10) (Figure 3b).

### 2.4. Circulating Levels of miR-27a-3p, miR-29b-3p, miR-122-5p, and miR-222-3p

**Figure 4:**
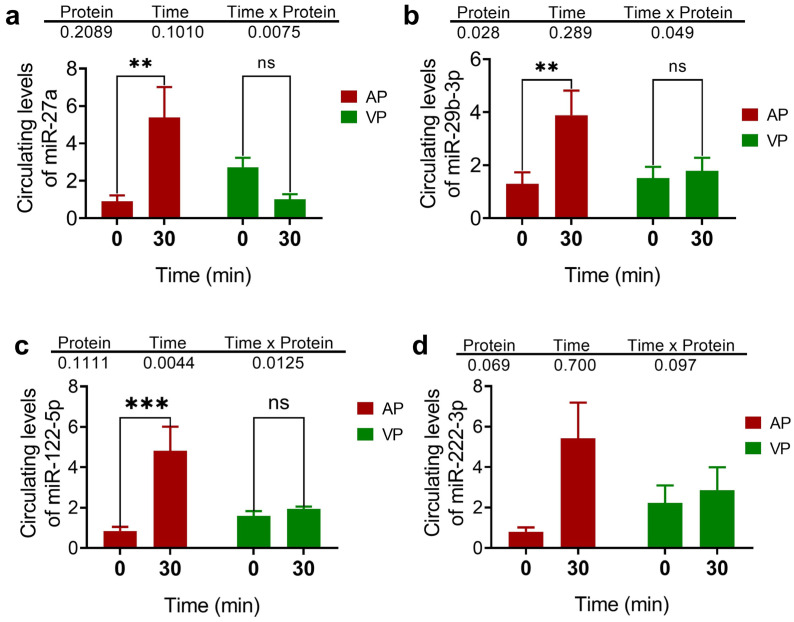
Circulating levels of miR-27a (**a**), miR-29b-3p (**b**), miR-122-5p (**c**), and miR-222-3p (**d**). The data are presented as the means ± SEs. Two-way ANOVA and post hoc Fisher’s LSD test were used to evaluate the differences in the relative expression of miRNAs between treatments (VP vs. AP) and time points (0 and 30 min). where ** *p* < 0.01, and *** *p* < 0.001. The abbreviation “ns” is not significant.

**Figure 5:**
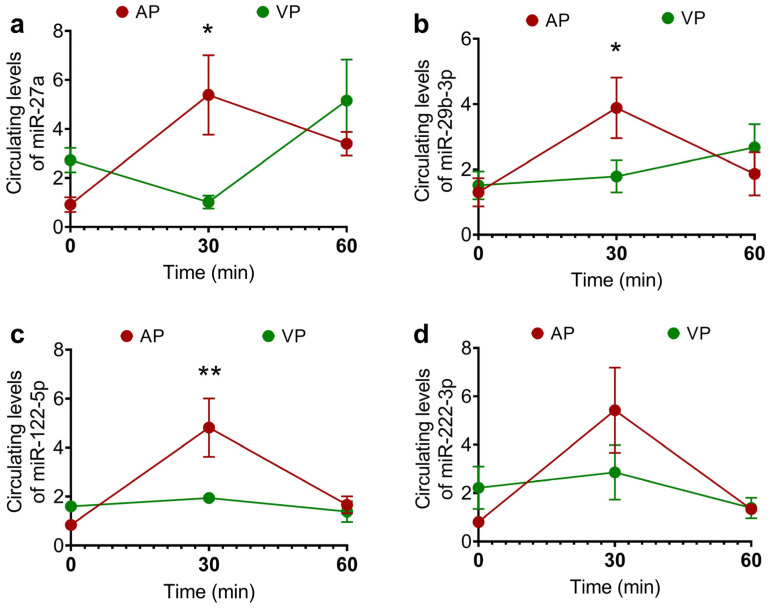
Circulating levels of miRNAs in the acute response 60 min after the consumption of a protein shake with calcium caseinate (AP) or soy protein isolate (VP): miR-27a (**a**), miR-29b-3p (**b**), miR-122-5p (**c**), and miR-222-3p (**d**). The data are presented as the means ± SEs, and the results of the statistical analysis were evaluated using two-way ANOVA, and a post hoc Sidak test was performed to evaluate the differences in the relative expression of miRNAs between treatments and time points. where * *p* < 0.05 and ** *p* < 0.01.

The effects of different types of protein on the concentrations of miRNAs were evaluated by measuring their plasma circulating levels. Compared with those at the baseline, the AP group presented increased circulating levels of miR-27a-3p, miR-29b-3p, and miR-122-5p (*p* < 0.05) (Figure 4a–c) and no differences in the expression of miR-222-3p with respect to the baseline were detected among the groups (Figure 4d). The subsequent analysis conducted over time at 0, 30, and 60 min revealed the same pattern and differences between treatments (Figure 5).

## 3. Discussion

This study demonstrated the effects of acute stimulation with different proteins on subjects with obesity and IR. To our knowledge, this study provides the first evidence showing dietary protein–metabolism–miRNA interactions. This interaction may be due to metabolic regulation through the ingestion of different types of protein, the metabolic response, and interactions at the molecular level between miRNAs. The results showed that the intake of VP in subjects with obesity and IR produced a smaller increase in concentrations of insulin, branched-chain amino acids and gluconeogenic amino acids and tended to upregulate the expression of exosomal microRNAs compared to the intake of AP.

AP induced a greater increase in insulin levels, surprisingly, without modifying glucose levels, which could be explained by three factors. First, amino acids are known insulin secretagogues [16,17,18,19], especially leucine, as a BCAA [20,21,22]. AP had a relatively high content of BCAAs, which was reflected in increased plasma BCAA levels, which could explain the increased insulin secretion. Second, as participants are insulin-resistant, glucose uptake by peripheral tissues, such as muscle or adipose tissue, is delayed, which is reflected in the lack of change in glucose levels despite the increase in insulin levels [23,24]. Third, an elevated insulin level also inhibits glucagon secretion and hepatic glucose production [25,26]. However, these regulatory mechanisms are altered in the insulin-resistant state [27,28,29,30].

Insulin secretion or decreased insulin sensitivity plays a key role in the pathogenesis of T2DM. Therefore, the search for optimal treatment and prevention strategies for this pathology is highly important. Glucose and insulin metabolism are mediated by complex processes related to various stimuli, such as protein sources. Interestingly, a study has shown a reduction in IR after the consumption of a high-protein diet compared with a reference diet [31]. In this regard, considering not only an increase in protein intake but also the source of the protein is important. The insulin concentration is modulated depending on the type of amino acids present in the dietary protein. For example, the plasma amino acid profile and concentration observed after casein consumption increases insulin secretion and PPARγ and GLUT2 expression in pancreatic islets compared with the changes induced by the plasma amino acid profile and concentration observed after soy protein consumption, indicating that a specific pattern of amino acids modulates glucose-stimulated insulin secretion by β-cells in the pancreas [32,33]. These amino acids can also modify insulin sensitivity, as they also modulate signalling pathways that can affect insulin action at the cellular and molecular levels via mTOR (mammalian target of rapamycin) by controlling components of the translational machinery, including initiation and elongation factors [34]. Other researchers have also previously shown that the type of dietary protein can determine circulating amino acid levels [8], which, in turn, may modulate insulin secretion [35,36].

This finding is very interesting since our results showed that VP consumption resulted in a lower increase in insulin levels than AP consumption. This evidence is consistent with a previous study showing that postprandial insulin levels increased after whey protein consumption [37]. Insulin–glucagon hormonal regulation has also been shown to be impaired under IR conditions [38]. However, our results revealed no differences between the groups. Both groups maintained elevated glucagon levels that did not seem to respond to the suppressive action of insulin; in contrast, a clear tendency was observed for the AP group to maintain a state of pronounced hyperinsulinaemia up to the middle of the curve. In people with obesity, hyperglucagonemia co-occurs with hyperinsulinaemia. This finding confirms an anabolic state in obesity, a condition that is driven by insulin, which is a hormone that is secreted in a greater proportion than glucagon, even in the fasting state. The hyperglucagonaemia observed in our study is a consequence of the decreased sensitivity of alpha cells to insulin, resulting in insulin not adequately suppressing glucagon secretion, as has been described by many researchers involved in a similar field of study [39].

In addition to the increase in insulin levels in the AP group, an increase in the circulating concentration of BCAAs was also observed compared with that in the VP group. One of the main amino acids in animal proteins is BCAAs, which represents almost 20% of dietary proteins [40]. Thus, the consumption of this type of protein could contribute to changes in plasma BCAA levels, which coincided with the changes in insulin levels in the AP group. Insulin can attenuate plasma BCAA levels in healthy, insulin-sensitive individuals with obesity [41]. This change may be because the ability of adipose tissue to degrade BCAAs decreases under conditions of chronic energy excess, leading to the accumulation of BCAAs and related metabolites, which are associated with the development of metabolic diseases [42]. Increased BCAA concentrations can activate pathways that promote IR through mTOR activation [43]. However, these conditions could be modulated by the consumption of plant protein, as demonstrated by our results, which show that, compared with the AP group, the VP group presented a lower increase in insulin levels, the sum of BCAA levels and the concentration of each BCAA in subjects with obesity and IR.

No significant differences were observed in the AUCs of the different amino acids after acute protein interventions. However, the concentrations of amino acids, such as valine, isoleucine, leucine, tyrosine, phenylalanine and tryptophan, were lower in the VP group than in the AP group at different time points in subjects with obesity and IR who consumed VP. This result is important, as the levels of the aromatic acids phenylalanine and tyrosine are elevated in obese subjects compared with lean subjects due to competition from neutral amino acids, including valine, isoleucine, leucine, tyrosine, phenylalanine and tryptophan, for entry into cells via the long-chain neutral amino acid transporter, and isoleucine, leucine, tyrosine, phenylalanine and tryptophan for entry into cells via the long-chain neutral amino acid transporter; thus, excess BCAAs may impair aromatic amino acid transport and alter circulating BCAA levels [7]. Another change in the VP group was an increase in the arginine concentration. In this context, although glucose-stimulated insulin secretion was impaired, as was the case in the population studied, this impairment could be regulated by arginine-stimulated insulin secretion [44]. However, studies have reported that the effect of arginine depends on the disease duration and drug treatment [45].

We evaluated the effects of protein intake not only on biochemical parameters but also at the molecular level. Importantly, to our knowledge, no evidence is available from clinical studies that evaluate how a dietary stimulus modulates the expression of miRNAs and how it impacts the pathophysiological state of individuals. Our results revealed that, in the AP group, an increase in miR-27a-3p expression was detected after 30 min, which coincided with the highest insulin concentration. According to a previous report, miR-27a participates in the regulation of genes involved in the insulin signalling pathway in models of obesity and after nutritional intervention. Previous studies have shown that serum miR-27a levels directly correlate with obesity and IR in a high-fat diet-induced animal obesity model [46]. They also showed the same association in a childhood population with obesity [47]. An in vitro study revealed that miR-27a mediated the repression of the 3’UTR of PPARγ, which is involved in regulating the insulin signalling pathway by participating in the PPARγ-PI3K/AKT-GLUT4 axis [48]. The pattern of miR-27a expression was similar to that of miR-29a and miR-122. Notably, miR-29a has also been reported to regulate insulin signalling through the regulation of PI3K [49]. The level of miR-29a is increased in the exosomes of adipose tissue macrophages from obese mice [50].

Although no significant differences in miR-222 expression were detected, differences in miR-122 expression were detected. miR-222 inhibits the expression of the phosphatase and tensin homologue (PTEN) in the insulin signalling pathway. PTEN phosphorylates phosphatidyl inositol 3-phosphate (PIP3) to activate the PI3K/Akt complex. Failure to activate this complex prevents the translocation of GLUT4 to the plasma membrane, disrupting glucose entry into the cell [51]. Both miRNAs are widely associated with obesity or the risk of associated metabolic conditions [14]. González-Arce et al. [14] reported the increased expression of miR-122 and miR-222 in a group of Mexican Mayan children with obesity compared to a group of children with normal weight. A positive correlation was also detected between high protein intake and increased miR-222 expression [14]. Although our study revealed no differences in miR-222 expression between the intervention groups, importantly, this study provides the first evidence showing the dynamics of miRNA expression during the hours after an acute dietary stimulus.

The present study has several limitations, one of which is the limited sample size for detecting treatment differences. However, this study is one of the first that aims to determine possible metabolic regulation through the ingestion of different types of protein, metabolic responses, and interactions at the molecular level with miRNAs, which runs the risk of having limited efficacy in detecting treatment differences. Another limitation we should mention is that we did not use an ultrasensitive kit for glucagon determination, which would have allowed us to identify subtle changes in serum levels after an acute stimulus, such as protein loading. Nonetheless, the utility it offers is the determination of the potential directionality of treatment differences that, probably with a larger number of patients, could allow a clear difference in miRNA expression between the study groups to be shown. In addition, the combination of a control group without obesity and IR could increase the difference in miRNA expression between the intervention groups treated with different protein sources.

## 4. Materials and Methods

### 4.1. Subjects

Subjects with obesity and IR were included. Subjects were recruited through advertisements on social networks. The inclusion criteria were adults aged 18 to 60 years with a diagnosis of obesity according to body mass index (BMI ≥ 30 kg/m^2^) and with IR according to the Homeostasis Model Assessment of IR (HOMA-IR) ≥ 2.5 [52]. The exclusion criteria were a diagnosis of any chronic disease, pregnancy, smoking status, chronic use of medications, and use of protein supplements. The study was approved by the Ethics Committee of Instituto Nacional de Ciencias Médicas y Nutrición Salvador Zubirán (REF 2373), registered at www.clinicaltrials.gov https://clinicaltrials.gov/study/NCT03627104?term=NCT03627104&rank=1&tab=table1 (accessed on 13 July 24) (NCT03627104) and conducted in accordance with the Declaration of Helsinki. Written informed consent was obtained from all participants.

### 4.2. Study Design

A clinical trial was conducted to evaluate the acute effects of different sources of dietary protein. The interventions included the consumption of a drink made from vegetable protein (soy protein isolate) or animal protein (calcium caseinate) in a single dose (1 g/kg body weight protein). All participants consumed both interventions at different times, with a one-week washout between interventions. To prepare the shake mixture, 50 mL of purified water was added to every 5 g of protein powder; no additives or flavourings were added. These shakes were consumed in a single dose.

### 4.3. Anthropometric, Clinical, and Body Composition Parameters

The anthropometric, clinical, and body composition parameters of the participants were evaluated. Weight and body composition data were collected with an Inbody 720 multifrequency bioimpedance body composition analyser. (Bioespace Co., Seoul, Republic of Korea). Waist circumference was measured using the Lohman technique [53] with an SECA flexible tape measure (SECA, Hamburg, Germany). The height of barefoot participants positioned in the Frankfort plane was determined with a BSM370 automatic stadiometer (Bioespace Co., Ltd., Seoul, Republic of Korea). Blood pressure was determined with a digital blood pressure monitor (Omron, HEM-781INT, Shanghai, China).

### 4.4. Blood Samples

Blood samples were collected at baseline with a fasting period of 12 h for each intervention and then at 0, 15, 30, 30, 45, 45, 60, 90, 90, 120, 150 and 180 min after the participants consumed the beverage. The blood was centrifuged at 3000 rpm for 10 min, and plasma was obtained. The samples were stored at −80 °C until analysis.

### 4.5. Serum Biochemical Parameters

The basal serum concentrations of glucose, total cholesterol, LDL cholesterol, and triglycerides were determined using the enzymatic colorimetric method with a Cobas C111 Integra analyser from Roche^®^ Diagnostics, Indianapolis, IN, USA.

### 4.6. Glucose, Insulin, and Glucagon Kinetic Curves

Glucose, insulin, and glucagon concentrations were evaluated at 0, 15, 30, 30, 45, 60, 60, 90, 120, 150, and 180 min after the consumption of the beverage. Insulin and glucagon concentrations were measured by colorimetric enzymatic techniques using ELISA kits (80-INSHU-E01e ALPCO, Salem, MA, USA; 48-GLUHU-E01 ALPCO, Salem, MA, USA). The areas under the curves (AUCs) were subsequently calculated for glucose, insulin, and glucagon [54].

### 4.7. Plasma Amino Acid Profile

The amino acid profile was determined with high-performance liquid chromatography (HPLC). The plasma concentrations of amino acids were evaluated at 0, 15, 30, 45, 45, 60, 90, 120, 150, and 180 min after the consumption of the beverage. For the assay, 150 μL of plasma was added to 38 μL of sulfosalicylic acid (10%).

The samples were incubated for 30 min under refrigeration and then centrifuged at 14,000 rpm for 10 min at 4 °C. Afterwards, 100 μL of the supernatant was removed, and 1 μL of the internal standard (norvaline; 15 mM) was added; the sample was then derivatised and injected. The procedure was performed using a sampling device (Agilent; G1367F, Santa Clara, CA, USA) coupled to an HPLC (Agilent 1260 Infinity, Santa Clara, CA, USA) and a fluorescence detector (Agilent; G1321B, Santa Clara, CA, USA). A ZORBAX Eclipse AAA column was used and maintained at 40 °C. The chromatographic conditions were maintained according to the technical instructions of the column.

### 4.8. Analysis of Circulating MicroRNAs

The analysis of circulating miRNAs was performed using a quantitative polymerase chain reaction (qRT–PCR). The circulating microRNAs were isolated from plasma obtained at 0, 30, 60, 60, 90, 120, and 180 min after the consumption of the beverage. An exoRNeasy Midi Kit (Qiagen, Hilden, Germany; Catalogue No. 77144) was used. Quantification was performed using a Qubit microRNA Assay Kit (Invitrogen, Waltham, MA, USA). Reverse transcription (RT) of the extracted miRNAs was performed with the miRCURY LNA RT Kit (Qiagen, Hilden, Germany, Catalogue No. 339340). Subsequently, the expression of each miRNA (hsa-miR-27a-3p GeneGlobe ID YP00206038; hsa-miR-29b-3p GeneGlobe ID YP00204679; hsa-miR-122-5p GeneGlobe ID YP00205664; and hsa-miR-222-3p GeneGlobe ID YP00204551) was assessed via q-PCR using miRCURY LNA mIRNA PCR ASSAY (Qiagen, Hilden, Germany, No. Catalogue 339306). U6 (U6 snRNA (v2) GeneGlobe ID YP02119464) was used as an endogenous control. Determinations were performed in triplicate with a Roche^®^ LightCycler^®^ 480 thermal cycler (Roche Diagnostics, Rotkreuz, Switzerland). The expression of miRNAs was calculated using mean the threshold cycle delta–delta (ΔΔCt) method [55]. The baseline of the AP group was used as a reference.

### 4.9. Statistical Analysis

The distribution of quantitative variables was evaluated with the Shapiro–Wilk test. Continuous variables are presented as medians (IQRs 25–75) or means ± standard errors. Qualitative variables are presented as frequencies (%). The difference between quantitative variables was evaluated with the Mann–Whitney U test. The Friedman test was used to evaluate the differences in relative miRNA expression between treatments (VP vs. AP) and with respect to time (0 and 30 min). Two-way ANOVA and post hoc Fisher’s LSD test were used to evaluate the differences in the relative expression of miRNAs between treatments (VP vs. AP) and time points (0 and 30 min). Similarly, two-way ANOVA and post hoc Sidak tests were used to evaluate the differences in the relative expression of miRNAs between treatments and time points (0, 30 and 60 min). The results obtained were considered significant at a *p* value < 0.05. The data were analysed with SPSS for Windows (version 24, SPSS Inc., Chicago, IL, USA). Figures were generated with GraphPad Prism version 8 software.

## 5. Conclusions

In conclusion, our study demonstrates that an acute stimulus with different protein sources causes differences in insulin secretion and serum amino acid levels and that a dynamic response in miRNA expression appears over 3 h, providing evidence that circulating insulin levels may depend, in part, on the dietary protein source and the gene regulatory mechanisms that miRNAs participate in. However, further studies with a larger number of participants are needed to elucidate the medium-term effects of the consumption of different dietary protein sources on the expression of miRNAs related to IR in obese individuals.

## Data Availability

Data are available upon request to the corresponding author.

## Supplementary Materials

The following supporting information can be downloaded at https://www.mdpi.com/article/10.3390/ijms25147716/s1.
