# Supplementary material for: Acute Effects of Dietary Protein Consumption on the Postprandial Metabolic Response, Amino Acid Levels and Circulating MicroRNAs in Patients with Obesity and Insulin Resistance
Source: Int J Mol Sci. 2024 Jul 14;25(14):7716. doi: 10.3390/ijms25147716 (PMC11276941; doi:10.3390/ijms25147716)
Supplement: Supplementary file 1 [file ijms-25-07716-s001.zip › ijms-3062235-supplementary.pdf]

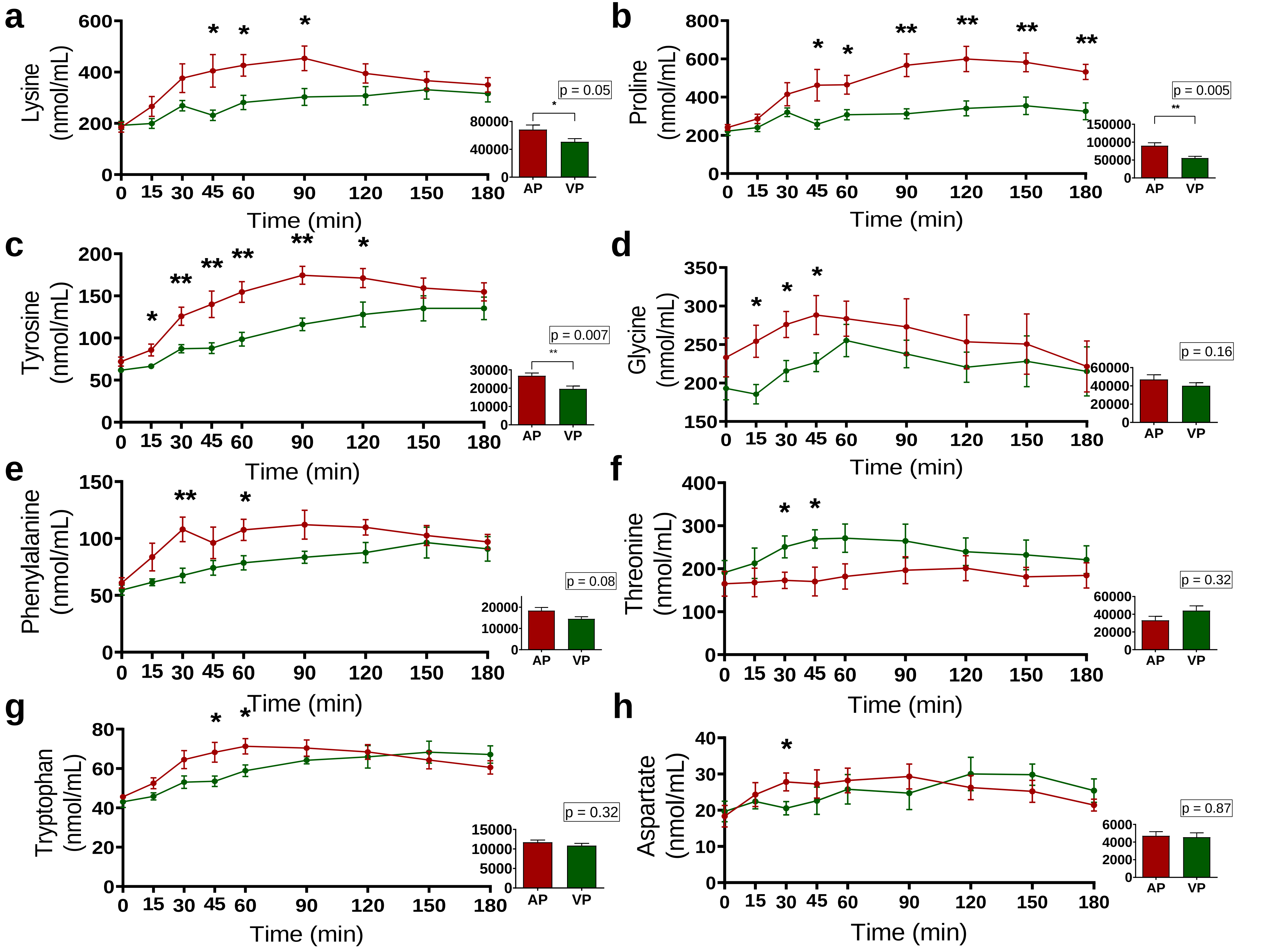

a
Lysine (nmol/mL)
Time (min)
p = 0.05
AP
VP
b
Proline (nmol/mL)
Time (min)
p = 0.005
AP
VP
c
Tyrosine (nmol/mL)
Time (min)
p = 0.007
AP
VP
d
Glycine (nmol/mL)
Time (min)
p = 0.16
AP
VP
e
Phenylalanine (nmol/mL)
Time (min)
p = 0.08
AP
VP
f
Threonine (nmol/mL)
Time (min)
p = 0.32
AP
VP
g
Tryptophan (nmol/mL)
Time (min)
p = 0.32
AP
VP
h
Aspartate (nmol/mL)
Time (min)
p = 0.87
AP
VP

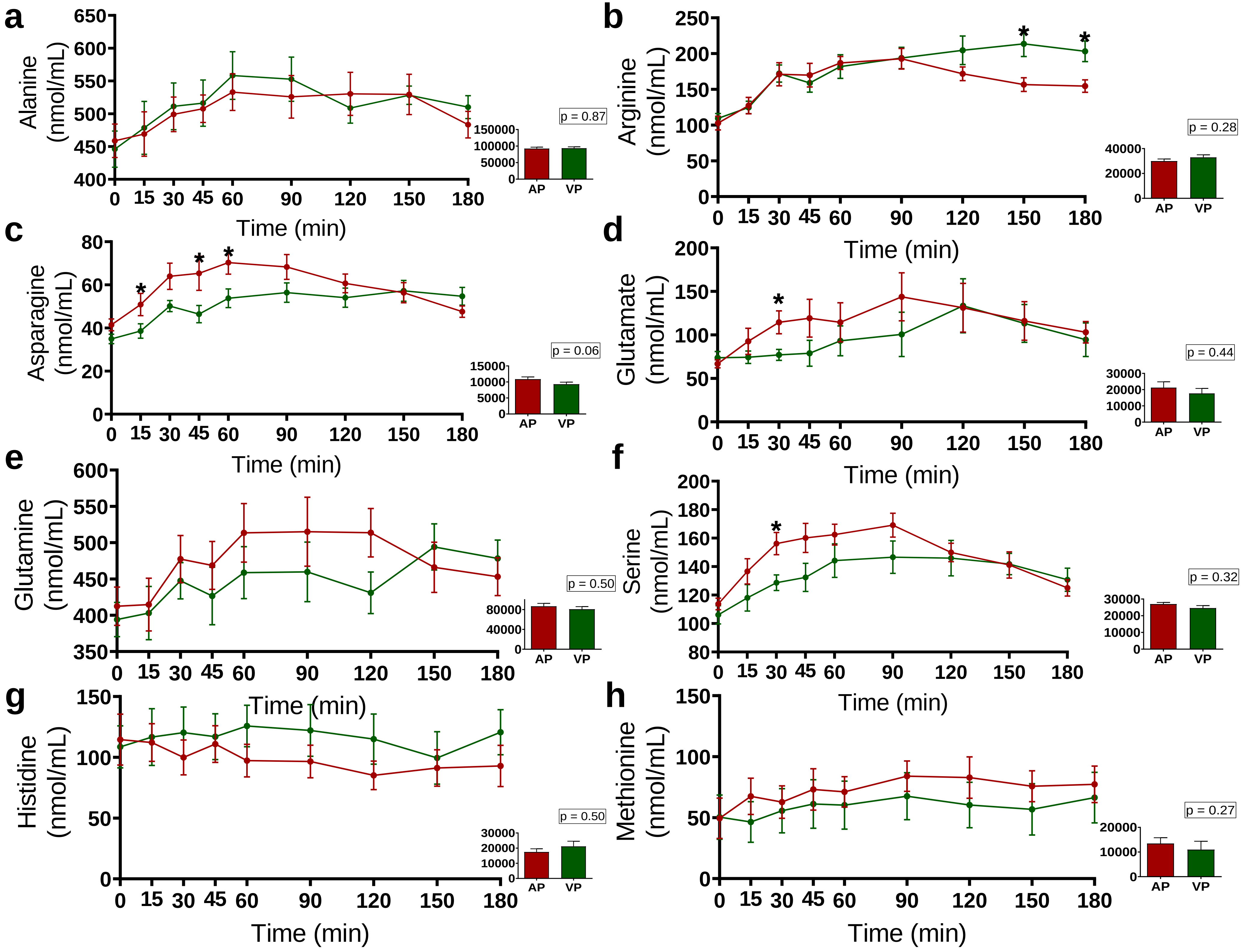

a
Alanine (nmol/mL)
Time (min)
p = 0.87
AP
VP
b
Arginine (nmol/mL)
Time (min)
p = 0.28
AP
VP
c
Asparagine (nmol/mL)
Time (min)
p = 0.06
AP
VP
d
Glutamate (nmol/mL)
Time (min)
p = 0.44
AP
VP
e
Glutamine (nmol/mL)
Time (min)
p = 0.50
AP
VP
f
Serine (nmol/mL)
Time (min)
p = 0.32
AP
VP
g
Histidine (nmol/mL)
Time (min)
p = 0.50
AP
VP
h
Methionine (nmol/mL)
Time (min)
p = 0.27
AP
VP

Supplementary table s1. Anthropometric, clinical and anthropometric characteristics of participants.

**Characteristics**	**n=10**
Age (years)	33.0 (27.5, 43.2)
Sex (n, %) Female	6 (60%)
Body weight (kg)	100(80.1,124)
BMI (kg/m²)	36.8 (31.8,41.3)
Waist (cm)	118(103,124)
Fat free mass (kg)	53.7 (43.1, 69.9)
Fat mass (kg)	44.4 (37.0, 53.9)
SBP (mmHg)	117 (112, 126)
DBP (mmHg)	80.0 (74.2,82.2)
Glucose (mgl/dL)	98.0 (93.3,105)
Total Cholesterol (mg/dL)	163 (155,183)
Tryglicerides (mg/dL)	155 (109,280)
Insulin (UI/mL)	16.2 (11.3, 19.6)
HOMA-IR	4.34 (3.10, 5.45)

Data are expresed as median (IQR 25,75). BMI: Body mass index; SBP: Systolic blood pressure; DBP: Diastolic pressure.

Figure legends

Supplementary Figure S1. Acute response of amino acids after 3 h of consuming protein shake made with calcium caseinate (PA) or soy protein isolate (PV) and the area under the curve (AUC), valine (A), isoleucine (B), leucine (C), lysine (D), proline (E), tyrosine (F), glycine (G), phenylalanine (H); data are presented as mean ± SE, statistical analysis was evaluated by Mann-Whitney U test, where $^{*}P < 0.05$ and $^{**}P < 0.01$.

Supplementary Figure S2. Acute response of amino acids after 3 h of consuming protein shake made with calcium caseinate (PA) or soy protein isolate (PV) and the area under the curve (AUC), alanine (A), arginine (B), asparagine (C), glutamate (D), glutamine (E), serine (F), histidine (G), methionine (H), threonine (I), tryptophan (J); data are presented as mean ± SE, statistical analysis was evaluated by Mann-Whitney U test, where $^{*}P < 0.05$ and $^{**}P < 0.01$.
